# An investigation of the use of acupuncture in stroke patients in Taiwan: a national cohort study

**DOI:** 10.1186/s12906-016-1272-0

**Published:** 2016-08-26

**Authors:** Shu-Wen Weng, Ta-Liang Chen, Chun-Chieh Yeh, Chien-Chang Liao, Hsin-Long Lane, Jaung-Geng Lin, Chun-Chuan Shih

**Affiliations:** 1 Graduate Institute of Chinese Medicine, College of Chinese Medicine, China Medical University, Taichung, 404 Taiwan; 2 Department of Chinese Medicine, Taichung Hospital, Ministry of Health and Welfare, Taichung 403, Taiwan; 3 Department of Anesthesiology, Taipei Medical University Hospital, Taipei, 110 Taiwan; 4 Department of Anesthesiology, School of Medicine, College of Medicine, Taipei Medical University, Taipei 110, Taiwan; 5 Health Policy Research Center, Taipei Medical University Hospital, Taipei, 110 Taiwan; 6 Department of Surgery, China Medical University Hospital, Taichung, 110 Taiwan; 7 Department of Surgery, University of Illinois, Chicago, Illinois USA; 8 School of Chinese Medicine for Post-Baccalaureate, College of Medicine, I-Shou University, Kaohsiung City, 824 Taiwan; 9 Department of Healthcare Administration, Asia University, Taichung, 413 Taiwan; 10 Ph.D. Program for the Clinical Drug Discovery from Botanical Herbs, College of Pharmacy, Taipei Medical University, Taipei 110, Taiwan

**Keywords:** Acupuncture, Complementary and alternative medicine, Stroke, Traditional Chinese medicine, Use

## Abstract

**Background:**

Acupuncture is considered a complementary and alternative medicine in many countries. The purpose of this study was to report the pattern of acupuncture use and associated factors in patients with stroke.

**Methods:**

We used claims data from Taiwan’s National Health Insurance Research Database and identified 285001 new-onset stroke patients in 2000–2008 from 23 million people allover Taiwan. The use of acupuncture treatment after stroke within one year was identified. We compared sociodemographics, coexisting medical conditions, and stroke characteristics between stroke patients who did and did not receive acupuncture treatment.

**Results:**

The use of acupuncture in stroke patients increased from 2000 to 2008. Female gender, younger age, white-collar employee status, higher income, and residence in areas with more traditional Chinese medicine (TCM) physicians were factors associated with acupuncture use in stroke patients. Ischemic stroke (odds ratio [OR] 1.21, 95 % confidence interval [CI] 1.15–1.28), having no renal dialysis (OR 2.76, 95 % CI 2.45–3.13), receiving rehabilitation (OR 3.20, 95 % CI 3.13–3.27) and longer hospitalization (OR 1.23, 95 % CI 1.19–1.27) were also associated with acupuncture use. Stroke patients using rehabilitation services were more likely to have more acupuncture visits and a higher expenditure on acupuncture compared with stroke patients who did not receive rehabilitation services.

**Conclusions:**

The application of acupuncture in stroke patients is well accepted and increasing in Taiwan. The use of acupuncture in stroke patients is associated with sociodemographic factors and clinical characteristics.

## Background

With the increasing use of complementary and alternative medicine (CAM) worldwide [1], the 1-year prevalence of CAM use in the United States and United Kingdom were found to be as high as 33.2 % and 26.3 %, respectively [2, 3]. The estimated out-of-pocket cost for CAM was $33.9 billion in the USA in 2007 [4]. Acupuncture is considered a subtype of traditional Chinese medicine (TCM) [5], which has been used for at least 2000 years in China, and it has gained attention in the United States since 1971. Currently, it is widely used in many countries [1, 5]. A cross-sectional survey showed that at least 1.5 % of adults had used acupuncture in the past 12 months in the United States in 2007 [6].

Stroke remains the leading cause of adult disabilities worldwide, with an estimated direct medical cost of $20.6 billion in the United States in 2010 [7]. The 1-year cost of stroke ranges from $7,342 to $146,149 per patient in several countries [8]. Pneumonia, urinary tract infection, pain, dysphagia, depression, and recurrent stroke are common complications after stroke [9–11].

Acupuncture has been accepted in stroke rehabilitation in many countries, and the treatment is relatively safe and effective in improving post-stroke chronic symptoms, such as disability, shoulder pain, and dysphagia [5, 12–15]. In the United States, acupuncture was used more frequently in stroke compared with non-stroke patients [16]. A previous study also showed the high use of TCM among stroke patients in Taiwan [17]. However, limited information is available on the pattern of use of acupuncture in stroke patients.

Using the National Health Insurance Research Database, we conducted a nationwide, population-based cohort study to evaluate the pattern of acupuncture use in stroke patients. Another purpose of this study was to report the factors associated with acupuncture use among adult stroke patients.

## Methods

### Source of data

Since 1996, all medical claims of insured beneficiaries have been documented in the National Health Insurance Research Database, which was established by Taiwan’s National Health Research Institute. Information available for this study included gender, birth date, disease codes, health care rendered, medicines prescribed, diagnoses at admission and discharge, and medical institutions and physicians providing services. This study employed the All Stroke Database, which consisted of all prevalent and incident stroke patients across Taiwan between 2000 and 2008 [17–19].

### Ethical statement

Insurance reimbursement claims used in this study were obtained from Taiwan’s National Health Insurance Research Database, which is available for academic access. This study was conducted in accordance with the Helsinki Declaration. To protect personal privacy, the electronic database was decoded with patient identifications scrambled for further public access for research. Although the National Health Research Institute regulations do not require informed consent due to the use of decoded and scrambled patient identification, this study was approved by Taiwan’s National Health Research Institute (NHIRD-100-122) and the Institutional Review Board of E-DA Hospital, Kaohsiung, Taiwan (2014012) [17–19].

### Study design and population

We identified 285001 newly diagnosed, hospitalized stroke patients aged ≥20 years between 2000 and 2008 as our eligible study subjects from 23 million people allover Taiwan. Those with a previous stroke according to a physician’s diagnosis were excluded until 1996. To confirm that all stroke patients in our study were incident cases, only new-onset stroke cases were included. The outcome of this study was the prevalence of acupuncture use in people with a new diagnosis of stroke in the first year. This study compared sociodemographic factors, coexisting medical conditions, and stroke characteristics between stroke patients who used and did not use acupuncture.

### Criteria and definition

We defined stroke according to the International Classification of Diseases, 9th Revision, Clinical Modification (ICD-9-CM 430–438). Coexisting medical conditions included diabetes mellitus (ICD-9-CM 250), hypertension (ICD-9-CM 401–405), hyperlipidemia (ICD-9-CM 272.0–272.4), and myocardial infarction (ICD-9-CM 410 and 412). According to the administration codes (D8, D9) from reimbursement claims, regular renal dialysis (including hemodialysis and/or peritoneal dialysis) was also considered a coexisting medical condition among stroke patients in this study. We classified the frequency of acupuncture visits into quartiles. Stroke patients in the highest quartile of acupuncture visits were defined as high acupuncture users. Medical expenditures on acupuncture were also classified into quartiles. Stroke patients who were in the highest quartile of acupuncture expenditure were considered high acupuncture expenditure patients.

As Taiwan has 359 townships and city districts, we calculated the population density (persons/km^2^) of each of these administrative units. Based on the population density, these units were stratified into tertiles to designate areas of low, moderate, and high urbanization. We calculated the density of traditional Chinese physicians (traditional Chinese physicians/10,000 persons) based on the number of traditional Chinese physicians per 10,000 residents in each administrative unit. The first, second, and third tertiles were considered areas with low, moderate, and high physician densities, respectively. Based on the Ministry of Health and Welfare criteria, low income status was defined as qualification for waived medical co-payments.

### Statistical analysis

To observe the trend of acupuncture use, the annual prevalence of stroke patients using acupuncture treatment was calculated from 2000 to 2008. We used chi-square tests to compare the difference in sociodemographics, coexisting medical conditions, and characteristics of hospitalization between stroke patients who did and did not use acupuncture. Univariate and multivariate logistic regression analyses were performed to calculate crude and adjusted odds ratios (ORs) and 95 % confidence intervals (CIs) that measured the relationships between acupuncture use and associated characteristics in stroke patients. These characteristics included sex, age, income status, occupation, urbanization, density of traditional Chinese physicians in the area, disease history, use of rehabilitation, type of stroke, and in-hospital characteristics. All analyses were performed using Statistical Analysis Software version 9.1 (SAS Institute Inc., Cary, North Carolina, USA). A two-sided probability value of <0.05 was considered statistically significant.

## Results

**Fig. 1:**
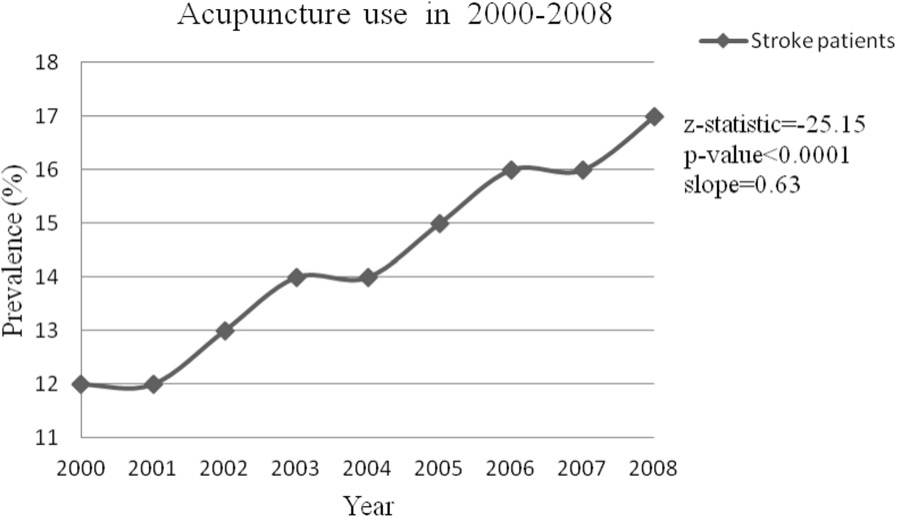
Prevalence of Acupuncture use among stroke patients in Taiwan from 2000–2008 (*Cochran-Armitage Trend Test)

The prevalence of acupuncture use among stroke patients increased from 12 % in 2000 to 17 % in 2008 (*p* < 0.0001) (Fig. 1). A higher incidence of acupuncture use was found in men, younger patients, higher income people, white-collar employees, residents living in highly urbanized areas, and areas with more TCM physicians.

The multivariate logistic regression analysis yielded the ORs of factors associated with acupuncture use in stroke patients (Tables 1 and 2), including female gender (OR 1.04, 95 % CI 1.01–1.06), age 30–39 years (OR 4.05, 95 % CI 3.77–4.36), very high income status (OR 1.55, 95 % CI 1.47–1.65), white-collar employee status (OR 1.16, 95 % CI 1.12–1.20), residence in highly urbanized areas (OR 1.44, 95 % CI 1.37–1.52), residence in areas with more TCM physicians (OR 1.43, 95 % CI 1.39–1.48), and use of other types of rehabilitation (OR 3.20, 95 % CI 3.13–3.27). Acupuncture users also experienced greater incidences of hypertension (OR 1.18, 95 % CI 1.15–1.21) and hyperlipidemia (OR 1.30, 95 % CI 1.26–1.35) but lower incidences of myocardial infarction (OR 1.18, 95 % CI 1.09–1.28) and renal dialysis (OR 2.76, 95 % CI 2.45–3.13). Ischemic stroke (OR 1.21, 95 % CI 1.15–1.28) and longer hospitalization (OR 1.23, 95 % CI 1.19–1.27) were also associated with acupuncture use.

**Table 1:** Comparison of sociodemographic characteristics between stroke patients with and without acupuncture treatment in 2000–2008

	Acupuncture use				*p*-value	OR	(95 % CI)*
	No (*N* = 242213)		Yes (*N* = 42788)				
Sex	n	(%)	n	(%)	<0.0001		
Women	101,397	(85.5)	17,225	(14.5)		1.04	(1.01–1.06)
Men	140,816	(84.6)	25,563	(15.4)		1.00	(reference)
Age, years					<0.0001		
20–29	1819	(83.1)	369	(16.9)		3.44	(3.04–3.89)
30–39	5680	(78.9)	1517	(21.1)		4.05	(3.77–4.36)
40–49	20,319	(79.3)	5298	(20.7)		3.79	(3.60–4.00)
50–59	39,190	(79.6)	10,054	(20.4)		3.56	(3.39–3.73)
60–69	58,684	(82.6)	12,374	(17.4)		3.05	(2.91–3.18)
70–79	74,891	(87.8)	10,424	(12.2)		2.04	(1.95–2.13)
≥ 80	41,630	(93.8)	2752	(6.2)		1.00	(reference)
Mean ± SD	67.4 ± 13.3		62.5 ± 12.5		<0.0001		
Income					<0.0001		
Very low	13,611	(86.1)	2203	(13.9)		1.00	(reference)
Low	71,645	(85.4)	12,283	(14.6)		1.23	(1.16–1.30)
Moderate	42,567	(84.9)	7565	(15.1)		1.31	(1.24–1.38)
High	88,550	(87.1)	13,152	(12.9)		1.30	(1.23–1.38)
Very high	25,840	(77.3)	7585	(22.7)		1.55	(1.47–1.65)
Occupation					<0.0001		
White collar	73,946	(82.0)	16,188	(18.0)		1.16	(1.12–1.20)
Blue collar	115,906	(86.5)	18,067	(13.5)		1.00	(reference)
Other	52,361	(86.0)	8533	(14.0)		1.06	(1.02–1.11)
Urbanization					<0.0001		
Low	17,323	(90.3)	1858	(9.7)		1.00	(reference)
Moderate	93,336	(87.4)	13,509	(12.6)		1.21	(1.15–1.28)
High	131,554	(82.8)	27,421	(17.3)		1.44	(1.37–1.52)
Density of TCM					<0.0001		
Low	64,074	(88.8)	8117	(11.2)		1.00	(reference)
Moderate	121,661	(84.6)	22,199	(15.4)		1.17	(1.14–1.21)
High	56,478	(81.9)	12,472	(18.1)		1.43	(1.39–1.48)
Mean ± SD	1.6 ± 1.2		1.8 ± 1.3		<0.0001		

*Logistic regression model included sociodemographics and medical conditions; Hosmer-Lemeshow goodness of fit, *p*-value = 0.0006; c-statistic = 0.71; CI, confidence interval; OR, odds ratio; TCM, traditional Chinese medicine

**Table 2:** Medical conditions of stroke patients with and without acupuncture treatment in 2000–2008

	Acupuncture						
	No (*N* = 285329)		Yes (*N* = 47832)		*p*-value	OR	(95 % CI)*
Rehabilitation					<0.0001		
No	166,526	(90.7)	17,087	(9.2)		1.00	(reference)
Yes	75,687	(74.7)	25,701	(25.3)		3.20	(3.13–3.27)
Diabetes					<0.0001		
No	172,698	(85.2)	29,983	(14.8)		1.00	(reference)
Yes	69,515	(84.4)	12,805	(15.6)		1.00	(0.98–1.03)
Hypertension					<0.0001		
No	93,695	(86.0)	15,290	(14.0)		1.00	(reference)
Yes	148,518	(84.4)	27,498	(15.6)		1.18	(1.15–1.21)
Hyperlipidemia					<0.0001		
No	219,658	(85.4)	37,503	(14.6)		1.00	(reference)
Yes	22,555	(81.0)	5285	(19.0)		1.30	(1.26–1.35)
MI					<0.0001		
No	236,962	(84.9)	42,037	(15.1)		1.18	(1.09–1.28)
Yes	5251	(87.5)	751	(12.5)		1.00	(reference)
Dialysis					<0.0001		
No	238,140	(84.9)	42,500	(15.1)		2.76	(2.45–3.13)
Yes	4073	(93.4)	288	(6.6)		1.00	(reference)
Type of Stroke					<0.0001		
Hemorrhage	53,950	(83.4)	10,714	(16.6)		1.02	(0.96–1.08)
Ischemia	173,573	(85.2)	30,155	(14.8)		1.21	(1.15–1.28)
Other	14,690	(88.5)	1919	(11.6)		1.00	(reference)
LOS, days					<0.0001		
1–5	84,534	(87.4)	12,172	(12.6)		1.00	(reference)
6–9	63,969	(85.9)	10,499	(14.1)		1.06	(1.03–1.09)
10–14	34,261	(83.8)	6605	(16.2)		1.16	(1.12–1.20)
15–19	16,537	(82.7)	3465	(17.5)		1.22	(1.17–1.28)
≥20	43,092	(81.1)	10,047	(18.9)		1.23	(1.19–1.27)
Mean ± SD	12.8 ± 17.5		14.9 ± 17.0		<0.0001		

*Logistic regression model included sociodemographics and medical conditions; Hosmer-Lemeshow goodness of fit, *p*-value = 0.001; c-statistic = 0.71; CI, confidence interval; LOS, length of stay; MI, myocardial infarction; OR, odds ratio

The average number of acupuncture visits in stroke patients was higher in males than in females (6.5 ± 7.8 vs. 6.4 ± 7.7, *p* < 0.001) (Tables 3 and 4). Stroke patients who had a low income, were white-collar employees, lived in highly urbanized areas and areas with more traditional Chinese physicians, used rehabilitation services, suffered from hemorrhagic stroke or ischemia, or had a longer hospitalization made more acupuncture visits. Patients who had more acupuncture treatment visits also had a higher expenditure on acupuncture.

**Table 3:** Post-stroke visits and expenditure on acupuncture treatment within one year in stroke patients in 2000–2008 by sociodemographics

		Medical visits		Medical expenditure	
	n	Mean ± SD	*p*-value	Mean ± SD	*p*-value
Sex			0.0155		0.0264
Women	17,225	6.4 ± 7.7		212 ± 258	
Men	25,563	6.5 ± 7.8		219 ± 271	
Age			0.0342		<0.0001
20–29	369	6.4 ± 7.0		219 ± 272	
30–39	1517	6.8 ± 7.8		240 ± 302	
40–49	5298	6.7 ± 8.2		226 ± 279	
50–59	10,054	6.5 ± 7.7		217 ± 267	
60–69	12,374	6.4 ± 7.6		210 ± 254	
70–79	10,424	6.4 ± 7.7		216 ± 263	
≥80	2752	6.2 ± 7.9		208 ± 267	
Income			<0.0001		<0.0001
Very low	12,172	5.0 ± 6.5		168 ± 220	
Low	10,499	5.7 ± 7.1		187 ± 234	
Moderate	6605	6.7 ± 7.9		217 ± 262	
High	3465	7.6 ± 8.4		246 ± 276	
Very high	10,047	8.5 ± 8.9		295 ± 321	
Occupation			<0.0001		<0.0001
White collar	16,188	6.9 ± 8.0		229 ± 276	
Blue collar	18,067	6.1 ± 7.4		203 ± 253	
Other	8533	6.6 ± 7.9		219 ± 269	
Urbanization			<0.0001		<0.0001
Low	1858	5.2 ± 6.8		173 ± 229	
Moderate	13,509	6.0 ± 7.4		202 ± 259	
High	27,421	6.8 ± 8.0		226 ± 270	
Density TCM			<0.0001		<0.0001
Low	8117	5.9 ± 7.2		198 ± 250	
Moderate	22,199	6.5 ± 7.8		215 ± 263	
High	12,472	6.7 ± 7.9		230 ± 279	

TCM, traditional Chinese medicine

**Table 4:** Post-stroke visits and expenditure on acupuncture treatment within one year in stroke patients in 2000–2008 by medical condition

		Medical visits		Medical expenditure	
	n	Mean ± SD	*p*-value	Mean ± SD	*p*-value
Rehabilitation			<0.0001		<0.0001
No	17,087	4.8 ± 6.3		157 ± 206	
Yes	25,701	7.6 ± 8.4		256 ± 292	
Diabetes mellitus			0.02		0.01
No	29,983	6.5 ± 7.8		218 ± 268	
Yes	12,805	6.3 ± 7.7		211 ± 259	
Hypertension			0.77		0.93
No	15,290	6.5 ± 7.8		216 ± 269	
Yes	27,498	6.5 ± 7.7		216 ± 263	
Hyperlipidemia			0.35		0.98
No	37,503	6.5 ± 7.8		216 ± 266	
Yes	5285	6.4 ± 7.6		216 ± 259	
MI			0.14		0.15
No	42,037	6.5 ± 7.8		216 ± 266	
Yes	751	6.1 ± 7.0		204 ± 240	
Renal dialysis			0.0002		<0.0001
No	42,500	6.5 ± 7.8		217 ± 266	
Yes	288	5.1 ± 6.1		161 ± 220	
Length of stay, days			<0.0001		<0.0001
1–5	12,172	5.0 ± 6.5		168 ± 220	
6–9	10,499	5.7 ± 7.1		187 ± 234	
10–14	6605	6.7 ± 7.9		217 ± 262	
15–19	3465	7.6 ± 8.4		246 ± 276	
≥20	10,047	8.5 ± 8.9		295 ± 321	
Type of Stroke			<0.0001		<0.0001
Hemorrhage	10,714	7.4 ± 8.5		253 ± 296	
Ischemia	30,155	6.3 ± 7.5		207 ± 256	
Other	1919	4.7 ± 6.3		154 ± 200	

MI, myocardial infarction

## Discussion

Our study found that the prevalence of acupuncture use among stroke patients significantly increased from 2000 to 2008 in Taiwan, and the sociodemographic characteristics were highly correlated with recent acupuncture use. In contrast to the previous report that was based on a cross-sectional sample [17], this study included all of Taiwan’s stroke patients and evaluated the patterns of acupuncture use.

The incidence of acupuncture use in the general population has been reported in the United States as 4.1 % in 2002 and 6.8 % in 2007 [6, 20]. The increasing use of acupuncture was investigated in the western countries [6, 20]. However, Chinese herbal medicine is not common in western countries. In Taiwan, Chinese herbal medicine and acupuncture were covered in the traditional Chinese medicine which is commonly used. Frequent use of acupuncture treatment was found in people with chronic diseases, such as osteoarthritis [21], cancer [22], and stroke [16]. The use of CAM is a trend, and it is not surprising that our study found that the use of acupuncture in stroke patients increased from 12 % in 2000 to 17 % in 2008. Demographic factors, such as age and sex, are associated with the patient’s choice of acupuncture [20, 23]. Compared with men, women were more likely to use acupuncture in this study. Several surveys also showed similar findings, namely, women had a higher use of TCM than men [17, 24]. Younger stroke patients were more likely to use acupuncture than older patients in this study. The association between young or middle age and acupuncture use was investigated in previous studies [17, 20, 23, 24]. The finding of better functional outcomes in younger stroke patients is not unexpected, as they had more home support and motivation [25]. A previous study suggested that young people seek more effective ways to improve their well-being and health and to relieve disease symptoms [26]. It is reasonable that younger stroke patients had a higher tendency to use acupuncture and to have a higher acupuncture expenditure than older patients in this study.

The increasing use of TCM is somewhat related to the growth of the number of TCM physicians in Taiwan [17]. A previous investigation reported that the density of TCM increased from 1.39 physicians per 10,000 residents in 1996 to 1.78 physicians per 10,000 residents in 2001 [27]. Our results also showed that the prevalence of acupuncture use in stroke patients increased with the increasing density of TCM physicians. High urbanization was associated with TCM use in a previous survey [17, 24]. Because acupuncture is a subtype of TCM, it is not surprising that we found in the present study that people who lived in an urbanized area were more likely to use acupuncture.

Economic growth is a determinant of physician supply and utilization of medical services [28]. In Taiwan, TCM has become an increasingly popular form of medicine, particularly after the implementation of the National Health Insurance in the medical care system since 1995. In this study, the frequency of acupuncture use and related insurance-paid expenditure were higher in stroke patients with a low income than in those without a low income. Co-payment is considered an important factor in the use of medical services [29]. According to the Ministry of Health and Wealth [17–19], patients with low-income status who do not need to pay a co-payment when receiving medical services may have more medical visits for acupuncture treatment than stroke patients with higher incomes.

Among the stroke patients, diabetes, hypertension, hyperlipidemia, myocardial infarction, and kidney insuffucuency were common coexisting medical conditions that were also considered comorbidities in this study [30]. Acupuncture treatment can lower blood pressure [31, 32]. Moreover, evidence-based studies have shown acupuncture’s beneficial effects in addressing physical illness in stroke patients [12–14, 33]. We found that patients with comorbidities of hypertension or hyperlipidemia were more likely to undergo acupuncture treatment. People with more chronic diseases were likely to use TCM, which was confirmed in previous surveys [17, 24].

In this study, we found that stroke patients who used acupuncture were more likely to simultaneously undergo conventional medical rehabilitation. Previous reports showed a high use of CAM in stroke survivors in many countries [16, 34]. Medical pluralism, such as adopting more than one medical system or the use of both conventional medicine and CAM for health and illness, is also common in Taiwan [35, 36].

Stroke patients with longer hospitalizations were more likely to undergo acupuncture treatment. Stroke patients who had a longer length of hospital stay may have more neurological impairment [37], so they may require more or longer rehabilitation for stroke. Our study showed that the frequency of acupuncture visits and related expenditure were higher in stroke patients after a longer length of hospital stay.

The incidence of ischemia is higher than that of other stroke subtypes [38]; however, subarachnoid hemorrhage resulted in a higher re-admission rate or mortality than ischemic stroke, and patients with a subarachnoid hemorrhage used more medical resources following stroke admission [39]. Conventional therapy with acupuncture treatment for acute ischemic stroke for four weeks has been shown to improve self-care ability and quality of life compared with sham acupuncture [40]. In this study, patients with ischemic stroke were more likely to use acupuncture than those with a subarachnoid hemorrhage. The frequency of acupuncture visits and related expenditures were higher in ischemic stroke patients.

This study had some limitations. First, we used retrospective medical claims data from health insurance claims data that lacked detailed patient information on clinical risk scores (e.g., National Institute of Health Stroke Scale score, Barthel index, and Rivermead index) and lifestyle, physical, psychiatric, and biochemical measures. We were unable to determine whether these factors were causally related to acupuncture use. Second, our study used ICD-9-CM codes claimed by physicians for the diagnosis of stroke without clarifying the severity of disease. Third, information on folk therapy was not available in the National Insurance Research Database. In addition, in-come measurement and out-come measurements of stroke patients are unavailable in this study, which are valuable indicators for efficacy of acupuncture treatment. It is also one of our study limitations. Finally, this study was based on cross-sectional analyses of acupuncture use in stroke patients. Understanding the benefit of acupuncture in stroke patients requires further cohort studies.

## Conclusions

In conclusion, the application of acupuncture in stroke patients is well accepted and increasing in Taiwan. The use of acupuncture in stroke patients is associated with sociodemographic factors and clinical characteristics.

## Abbreviations

CAM: Complementary and alternative medicine
CI: Confidence interval
ICD-9-CM: International classification of diseases, 9th revision, clinical modification
OR: Odds ratio
TCM: Traditional Chinese medicine
